# Dengue Virus Serotype 2 from a Sylvatic Lineage Isolated from a Patient with Dengue Hemorrhagic Fever

**DOI:** 10.1371/journal.pntd.0000423

**Published:** 2009-04-28

**Authors:** Jane Cardosa, Mong How Ooi, Phaik Hooi Tio, David Perera, Edward C. Holmes, Khatijar Bibi, Zahara Abdul Manap

**Affiliations:** 1 Institute of Health & Community Medicine, Universiti Malaysia Sarawak, Kota Samarahan, Sarawak, Malaysia; 2 Sibu Hospital, Sibu, Sarawak, Malaysia; 3 Center for Infectious Disease Dynamics, Department of Biology, The Pennsylvania State University, University Park, Pennsylvania, United States of America; 4 Fogarty International Center, National Institutes of Health, Bethesda, Maryland, United States of America; 5 University Clinic, Universiti Malaysia Sarawak, Kota Samarahan, Sarawak, Malaysia; University of California Berkeley, United States of America

## Abstract

Dengue viruses circulate in both human and sylvatic cycles. Although dengue viruses (DENV) infecting humans can cause major epidemics and severe disease, relatively little is known about the epidemiology and etiology of sylvatic dengue viruses. A 20-year-old male developed dengue hemorrhagic fever (DHF) with thrombocytopenia (12,000/ul) and a raised hematocrit (29.5% above baseline) in January 2008 in Malaysia. Dengue virus serotype 2 was isolated from his blood on day 4 of fever. A phylogenetic analysis of the complete genome sequence revealed that this virus was a member of a sylvatic lineage of DENV-2 and most closely related to a virus isolated from a sentinel monkey in Malaysia in 1970. This is the first identification of a sylvatic DENV circulating in Asia since 1975.

## Author Summary

Dengue viruses are mosquito-borne RNA viruses that cause a spectrum of illness from mild disease to life-threatening dengue hemorrhagic fever (DHF). Dengue viruses exist in two separate cycles in nature, circulating in either non-human primates or humans. The viruses that are endemic in humans today most likely evolved from non-human primate dengue viruses a few hundred years ago and have since established themselves as four distinct serotypes in human populations, causing periodic epidemics and severe disease. During the course of routine surveillance for dengue transmission in Malaysia during 2008, a sylvatic dengue virus type 2 was isolated from a young human male with DHF. The complete genome sequence of the virus was determined and found to be closely related to that isolated from a monkey in a similar region of Malaysia in 1970. This is the first sylvatic dengue virus isolated in Asia for more than 30 years.

## Introduction

Dengue viruses cause dengue fever (DF) and dengue hemorrhagic fever (DHF) in the tropics from Southeast and Southern Asia, the Caribbean, and many countries in South and Central America, and outbreaks are reported with increasing frequency globally. The four distinct dengue virus serotypes (DENV-1 to DENV-4) belong to the family *Flaviviridae* and are among the most important vector-borne pathogens of humans, causing up to 100 million cases annually.

Dengue viruses are mosquito-transmitted and circulate in both a sylvatic (enzootic) cycle involving non-human primates and various species of *Aedes* mosquito (such as *Ae. furcifer*, *Ae. luteocephalus* and *Ae. taylori*), and in a human (endemic) cycle principally vectored by *Aedes aegypti* [1],[2],[3]. The only sylvatic DENV serotype that has been isolated in Africa is DENV-2. In contrast, sylvatic DENV-1, DENV-2 and DENV-4 have been isolated in Asia [2], although the last isolation of a sylvatic virus (of DENV-4) occurred in 1975, and the ‘sylvatic’ isolate of DENV-1 is not phylogenetically distinct from human lineages so that its origin is uncertain. Phylogenetic data suggests that sylvatic DENV are the ancestors of those viruses that now circulate endemically in human populations [2]. Importantly, sylvatic dengue viruses have never been associated with major epidemics in humans [4]. Here we report a case of dengue hemorrhagic fever from whom we isolated a DENV-2 which was determined by phylogenetic analysis to be a sylvatic strain most closely related to a virus isolated by Rudnick from a sentinel monkey in peninsular Malaysia in 1970 [5].

## Materials and Methods

### The context

Our laboratory provides virology support in the state of Sarawak, Malaysia, part of which involves the isolation of virus from the blood of patients suspected of having DF or DHF. This is conducted as part of routine investigations for fever, which includes ruling out both dengue and malaria. Two primary care clinics and the university health centre act as sentinel clinics for public health surveillance to detect dengue in the community. During this routine surveillance program we isolated a sylvatic virus from a dengue febrile patient.

### Ethics statement

Further investigation of the virus isolated was performed after consultation with the patient and he has provided written consent to allow publication of his case.

### Virus isolation

Serum from the blood drawn at presentation was inoculated into C6/36 mosquito cells. When a cytopathic effect characteristic of DENV was noted, the culture was harvested and RNA extracted using the High Pure Nucleic Acid Extraction kit (Roche Diagnostics, Indianapolis, IN) and RT PCR performed to amplify a part of the NS5 gene using primers mFU1 and CFD2 as described by Chien and coworkers [6]. The amplicon was purified, sequenced and submitted to a BLAST search that returned a match to DENV-2. We then amplified the E/NS1 junction using primers AS2622DEN2 and S11871DEN2 [7] and used the sequence of this region to provide genotype information for this isolate, then designated DKD811.

The complete genome of isolate DKD811 was sequenced using overlapping degenerate primer sets spanning the whole genome that were designed using the Primaclade software [8] available at http://dousta.umsl.edu/cgi-bin/primaclade.cgi. A consensus sequence based on alignments of the complete genome sequences of DENV-2 sylvatic strains obtained from GenBank was used as the template for primer design. The PCR cycling conditions for all the overlapping primer sets included an initial denaturation step at 95°C for 5 minutes followed by 35 cycles of 95°C for 30 seconds, 55°C for 30 seconds and 72°C for 1 minute. A final extension at 72°C for 5 minutes was performed after the last cycle. The genome ends were sequenced using protocols for the Rapid Amplification of cDNA Ends (RACE) from Invitrogen (CA, USA). All amplified products were gel purified using the GENECLEAN III kit (BIO101, CA, USA) and were sequenced in both directions using the respective primers used to generate the PCR product. Sequencing was done using BigDye v3.1 (Applied Biosystems, CA, USA) and run on the 3130 Genetic Analyzer (Applied Biosystems, CA, USA).

### IgM capture ELISA (MAC ELISA)

The presence of dengue specific IgM was determined using a routine MAC ELISA described previously [9] for both dengue and Japanese encephalitis virus (JEV) run in parallel in order to discriminate DENV and JEV responses. Two sets of antigens were used: a mosquito cell derived cocktail of DENV-1, DENV-2, DENV-3 and DENV-4, and JEV separately. Specific responses to these antigens were determined using monoclonal antibodies to a dengue group reactive epitope and to JEV.

### Plaque reduction neutralization test (PRNT50)

The PRNT50 test was performed essentially as originally described by deMadrid and Porterfield [10] using PS Clone D cells and a semi solid carboxymethylcellulose overlay. Four fold serial dilutions of heat inactivated acute and convalescent sera from the patient taken 4 weeks apart, were incubated for 1 hour at 37°C in duplicate in 24 well plates. The cell suspension was then added and allowed to adhere at 37°C for 2 hours before addition of overlay. The plates were incubated for 5 days before the monolayer was stained and plaques counted.

### Phylogenetic analysis

To determine the evolutionary history of DKD811 we performed a phylogenetic analysis on 58 complete coding sequences of DENV-2 (total alignment length of 10,173 nt). Specifically, we inferred a Maximum Clade Credibility (MCC) tree using the Bayesian Markov Chain Monte Carlo (MCMC) method available in the BEAST package [11], thereby incorporating information on virus sampling time. This analysis utilized a strict molecular clock and a GTR+Γ model of nucleotide substitution for each codon position, although very similar results (with no major differences in topology or coalescent times) were obtained under a relaxed (uncorrelated lognormal) molecular clock and different substitution models (results available from the authors on request). As demographic history is a nuisance parameter in our study, we utilized the Bayesian skyline model as a coalescent prior. All chains were run for a sufficient length to ensure convergence with 10% removed as burn-in. This analysis also allowed us to estimate coalescent (divergence) times for each node on the DENV-2 phylogeny. The degree of uncertainty in each parameter estimate is provided by the 95% highest posterior density (HPD) values, while posterior probability values provide an assessment of the degree of support for each node on the tree.

## Results/Discussion

### The case

A 20-year old previously healthy male university student was admitted into a hospital in Sarawak (a Malaysian state on the island of Borneo) on January 3^rd^ 2008 for suspected dengue infection. He had returned to the university campus only two days before his hospital admission, having spent 4 weeks of end-of-semester vacation in peninsular Malaysia.

A day before his return to Sarawak, he fell ill with high fever and chills. He sought medical attention at the university health centre on day 4 of illness when his fever persisted with further signs and symptoms of increased fatigue, anorexia, recurrent vomiting and bouts of diarrhea. There was no history of cough, coryza, sore throat, conjunctivitis, skin rash, headache, arthralgia, myalgia, retro-orbital pain, bone pain, bleeding tendencies or abdominal pain. On examination, he looked lethargic, was febrile (37.5°C) and mildly dehydrated. No skin rashes or petechia were noted. The tourniquet test was negative. The other systemic examinations, including lung and abdomen, were unremarkable, and he was provided with symptomatic treatment. His blood investigation showed the following: hemoglobin 16.4 g/dL, hemotocrit 47.5%, white cell count 3200/uL (neutrophils 69.3%, lymphocytes 22.9%) and platelet count 81000/uL (Table 1).

**Table 1:** Laboratory investigations by day of illness.

	Day of Illness							
	Day 4	Day 5^a^	Day 6	Day 7	Day 8	Day 9^b^	Day 16	Day 37
Temperature	37.5	39.6	37	37	36.5	-	-	-
Hemoglobin (g/dL)	16.4	16.4	15.8	14.9	14.1	13.3	13.0	-
Total white cells (per uL)	3200	2600	4900	5300	4000	4400	5200	-
Platelets (per uL)	81000	32000	15000	12000	21000	53000	527000	-
Hematocrit (%)	47.5	50.9	48.3	44.3	43.7	40.1	39.3	-
Hemoconcentration (%)	20.9^c^	29.5^c^	22.9^c^	12.7	11.2	2.0	-	-
Lymphocytes (%)	22.9	-	-	-	-	-	42.9	-
Neutrophils (%)	69.3	-	-	-	-	-	48.8	-
Prothrombin (sec)	-	13.6	-	-	-	-	-	-
Partial Thromboplastin Time (sec)	-	61.6	-	-	-	-	-	-
Sodium (mmol/L)	-	128	126	-	-	-	-	-
Total Bilirubin (umol/L)	-	16	-	-	-	-	-	-
AST^d^ (U/L)	-	405	-	-	-	-	-	-
ALT^e^ (U/L)	-	194	-	-	-	-	-	-
Total protein (g/L)	-	83	-	-	-	-	-	-
Albumin (g/L)	-	46	-	-	-	-	-	-
Alkaline Phosphotase (U/L)	-	62	-	-	-	-	-	-
Virus isolation	DENV2	-	-	-	-	-	-	-
MAC ELISA								
Adj ODf DENV	0.202	-	-	-	-	-	-	0.818
Adj OD JEV	0.079							0.183
DENV cut off OD	0.380							0.380
JEV cut off OD	0.280							0.280
GAC ELISA^g^								
Adj OD DENV	0.564	-	-	-	-	-	-	1.140
Adj OD JEV	0.389							1.140
DENV cut off OD	0.400							0.400
JEV cut off OD	0.315							0.315

a Day of admission.

b Day of discharge.

c >20% hemoconcentration.

d Aspartate aminotransferase (AST).

e Alanine aminotransferase (ALT).

f Adjusted optical density (Adj OD).

g IgG-capture ELISA (GAC ELISA).

On the following day, he was admitted into hospital after his platelet count (performed at the university health centre) dropped further to 35000/uL. Examination at hospital admission revealed that he had facial flush and was dehydrated. His body temperature was 39.6°C, blood pressure was 112/72 mmHg and pulse rate was 81/min. Apart from mild hepatomegaly, the other systemic examinations, including cardiovascular and lung, revealed no abnormality. He was treated with intravenous fluid for rehydration. The results of laboratory investigations performed on admission and during his hospitalization are shown in Table 1. His fever resolved rapidly within 12 hours after hospital admission. Apart from having pruritic skin petechiae on his legs, there was no other spontaneous bleeding, overt clinical signs of plasma leakage such as pleural effusion, ascites or circulatory disturbance during the first 48 hours after defervescence. A blood investigation on day 16 of illness showed a platelet count of 527,000/uL and a hematocrit of 39.3%, indicating that during the acute infection the patient's hematocrit had risen by 29.5% above his baseline. These clinical features are consistent with DHF grade II [12].

IgM to DENV was absent in the first serum specimen but IgM seroconversion was demonstrated to DENV in the convalescent serum specimen taken a month after the first, confirming the diagnosis of DENV infection. DENV specific IgG was positive in the acute serum specimen and the convalescent specimen showed a high level of broadly reactive IgG to the flaviviruses DENV and JEV, suggesting a secondary immune response to DENV. Plaque reduction neutralization tests (PRNT50) with acute and convalescent sera against the patient's own DENV-2 isolate showed a seroconversion from <1∶10 to 1∶2560, confirming that this patient had an established DENV-2 infection.

### Evolutionary history of DKD811

**Figure 1:**
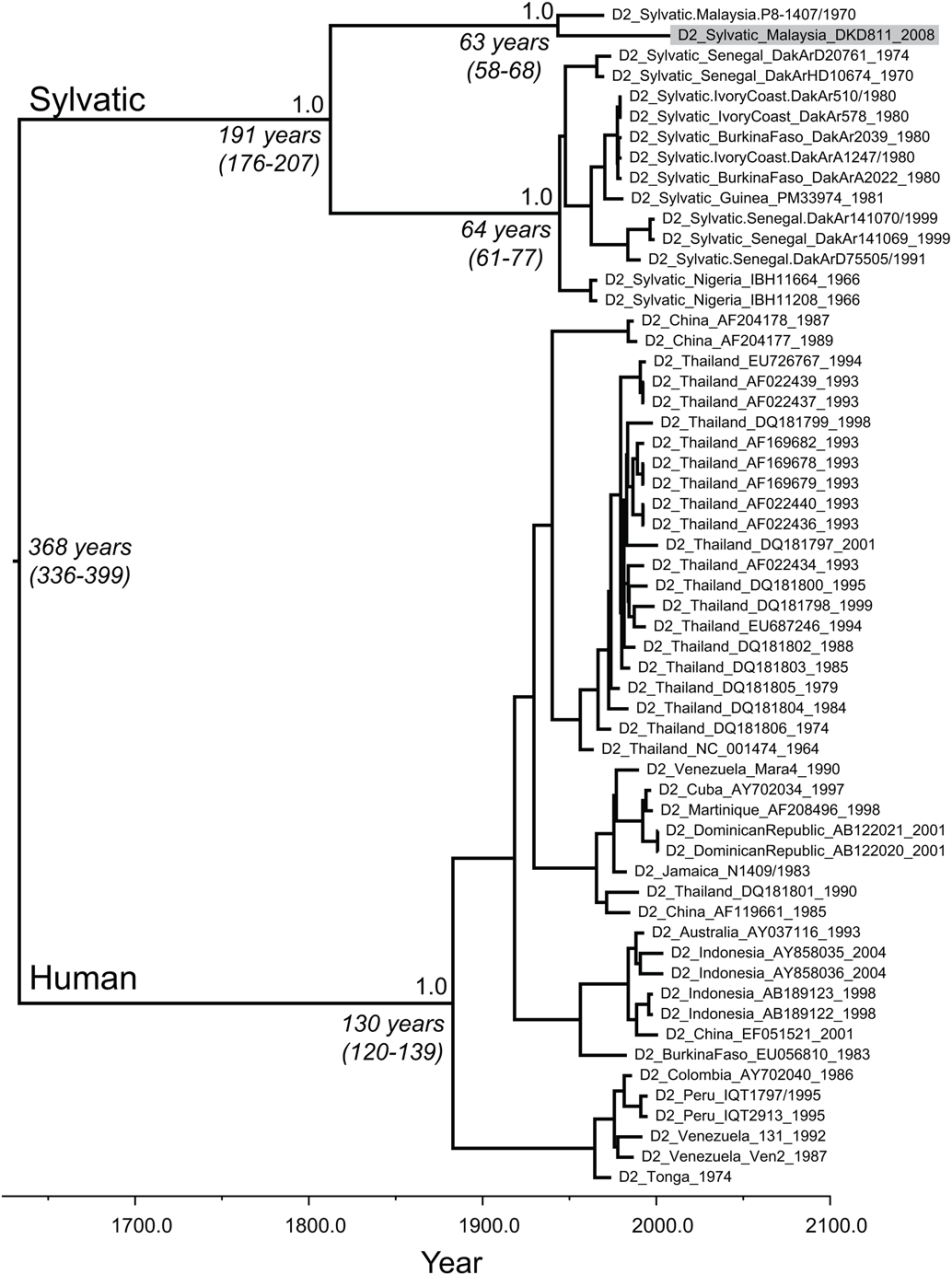
MCC tree of 58 complete coding sequences of DENV-2. Horizontal branches are drawn to a scale of estimated year of divergence with tip times reflecting sampling date (year). The tree is automatically rooted due to the assumption of a molecular clock. The coalescent (i.e. divergence) times of some key nodes, as well their 95% HPD values, are shown. Posterior probability values (all 1.0) are shown above nodes. Isolate DKD811 newly determined here is in the grey shaded box. Accession number for DKD811 full genome is FJ467493.

Our complete genome phylogenetic analysis clearly revealed that isolate DKD811 fell within the sylvatic lineage of DENV-2 and was most closely related to isolate P8-1407 sampled in Malaysia in 1970 (Figure 1). This phylogenetic history was supported by high posterior probability values (1.0). In addition, our molecular clock analysis suggested that these two Malaysian sylvatic viruses last shared a common ancestor approximately 63 years ago (95% HPD of 58–68 years), so that DENV-2 is likely to have been circulating in non-human primates in Malaysia for at least this time. Finally, this analysis suggested that the sylvatic and human lineages of DENV-2 last shared a common ancestor 368 years ago (95% HPD of 336–399 years, which is similar to previous estimates [13].

### Conclusion

Through a fever surveillance programme, we identified a patient with DHF grade II who had been infected with a sylvatic strain of DENV-2. This case is significant for several reasons. In particular, this is the first recorded isolation of a sylvatic DENV virus in Asia since 1975 and sylvatic isolates in general are characterized by their rarity. Given that DKD811 is most closely related to another Malaysia sylvatic virus (P8-1407) that was isolated in 1970, we surmise that DENV-2 was resident in non-human primates in this geographical location for the interim 38 years but without detection. Notably, our molecular clock analyses suggest that DENV-2 may been in Malaysian monkeys for at least 60 years.

It is also noteworthy that this sylvatic isolate of DENV-2 was associated with severe disease. Specifically, the virus was isolated from a single patient with DHF who had been on vacation in an area of the country where large numbers of dengue cases have been reported. Further, the sentinel monkey study site of Rudnick and colleagues was located in the same general area along the southern slopes of the Main Range in peninsula Malaysia, where the patient spent the 4 weeks prior to onset of illness. Although we were unable to ascertain if the sylvatic DENV-2 we isolated was circulating widely in the area our patient visited, the potential for a re-introduction of ancestral DENV2- into the human population should be evaluated [1].

This is the first report of a sylvatic dengue virus causing DHF, providing a unique opportunity to investigate whether this strain can cause DHF in non-human primates either as a primary or secondary infection. Non-human primates have been utilized to study some aspects of dengue infection, but in general the read-outs have been the level of viremia as well as other non-specific indicators of the establishment of an infection, together with the production of neutralizing antibodies [14]–[16]. In addition, the infecting viruses have not been of sylvatic origin. The identification of this sylvatic strain provides hope of being able to induce features of DHF such as thrombocytopenia, vascular leakage and hemoconcentration.
